# Emergency and persistence of *Escherichia coli* ST131 as community-onset antimicrobial resistant urinary tract infection in Rio de Janeiro, Brazil

**DOI:** 10.1016/j.bjid.2025.104555

**Published:** 2025-06-14

**Authors:** Eduardo Moreira de Castro, Isadora Silva Barcellos, Guilherme Santoro-Lopes, Ana Paula de Souza da Silva, Luís Guilherme de Araújo Longo, Mariana Anjo Barbosa, Gabriela Camarano de Oliveira, Lucas Cecílio Vilar, Adriana Lúcia Pires Ferreira, Karla Rodrigues Miranda, Beatriz Meurer Moreira

**Affiliations:** a Universidade Federal do Rio de Janeiro, Instituto de Microbiologia Paulo de Goes, Rio de Janeiro, RJ, Brazil; b Universidade Estácio de Sá, Faculdade de Medicina, Instituto de Educação Médica, Rio de Janeiro, RJ, Brazil; c Universidade Federal do Rio de Janeiro, Faculdade de Medicina, Rio de Janeiro, RJ, Brazil; d Diagnósticos da América S/A, DASA, Rio de Janeiro, RJ, Brazil

**Keywords:** Urinary tract infection, *Escherichia coli*, ST131, Antimicrobial resistance, Pandemic clones

## Abstract

Urinary Tract Infections (UTI) are among the most common public health problems worldwide, mostly caused by *Escherichia coli.* High-risk pandemic clones, especially ST131, are known for their association with multidrug resistance. A better understanding of epidemiologic and molecular characteristics may provide insights into the dissemination and evolution of this pathogen. The present study aims to investigate selected clonal characteristics of a large collection of UTI-causing *E. coli* isolates from Rio de Janeiro, an urban center in Brazil. We set up a collection of 992 *E. coli* isolates from patients with UTI in 2019. We determined antimicrobial susceptibility, Extended Spectrum Betalactamase (ESBL) production and clonal composition of isolates and compared results with data from 2015. Frequencies of four most isolated pandemic clones (ST131, ST69, ST73 and ST95) were determined by PCR; ST131 clades were determined by PCR and *fimH* gene sequence; ESBL-producing isolates underwent MLST. Resistance frequencies were > 30 % for ampicillin and ciprofloxacin. ST131 isolates were the most frequent clone (14 %), increasing significantly from 2015, comprising 52 % of all ESBL-producing strains. Clade C formed most ST131 isolates (56 %), including 40 % of all ESBL-producing isolates, most in Clade C2; almost all *fim*H30. ST131, formed by heterogeneous lineages, was established as a major source of ESBL isolates in the community, with a major contribution to antimicrobial resistant UTI.

## Introduction

Urinary Tract Infection (UTI), mostly caused by *Escherichia coli*, is a major source of outpatient visits worldwide.^1^, ^2^, ^3^ As a common type of community-acquired infection, UTI is responsible for the intensive use of antimicrobial agents in the community, contributing to the global spread of the serious public health problem of resistance.^4^ Indeed, the Global Antimicrobial Resistance and Use Surveillance System (GLASS) of 2022 showed a direct link between consumption and antimicrobial resistance.^5^

Reports of Multidrug-Resistant (MDR) isolates have increased over decades due to the spread of successful strains of extraintestinal *E. coli* (ExPEC).^6^, ^7^, ^8^, ^9^ Such clones are determined by Multilocus Sequence Typing (MLST) and *fim*H typing, among other techniques. Four pandemic lineages responsible for UTI are most frequently isolated worldwide: ST131, ST69, ST73, and ST95.^9^^,^^10^ While ST73 and ST95 are usually susceptible to several antimicrobial agents, ST69 and ST131 are highly resistant.^8^^,^^11^, ^12^, ^13^

The clonal characteristics of ST131 have been studied in detail due to particularly high rates of antimicrobial resistance and global dissemination.^8^ This clone has been associated with certain types of Extended-Spectrum Beta-Lactamases (ESBL), such as CTX-M-15^14^^,^^15^ and a selection of *fimH* gene alleles. ST131 is subdivided into clades and subclades A, B, C1, and C2, the last one associated with fluoroquinolone resistance and CTX-M production.^8^^,^^16^^,^^17^

Surveillance studies have shown that certain ExPEC clones are evolving, changing the population structure over time, with a potential impact on the pathogenic potential of UTI and antimicrobial resistance rates. A better understanding of these dynamics and the genetic makeup of pandemic lineages in successive collections of ExPEC over time may point ways to minimizing these threats.^18^ The frequency and extent of dissemination of *E. coli* pandemic strains in Brazil have been poorly studied.^11^^,^^19^, ^20^, ^21^, ^22^, ^23^ We had detected that the prevalence of ST131 as a community-onset UTI agent was rare (2 %, 3/139) during 2005–2006^19^ and increased to 9 % (45/499) in 2015.^11^ Moreover, resistance to CIP and broad-spectrum cephalosporins, absent from 2005 in ST131 isolates, reached 69 % (31/45) and 20 % (9/45), respectively, in 2015. The present study aims to investigate selected clonal characteristics of a large collection of UTI-causing *E. coli* isolates from Rio de Janeiro, a cosmopolitan urban center in Brazil, after the last assessment in 2015.

## Materials and methods

### Subjects, clinical specimens, and bacterial isolates

Urine specimens were obtained from outpatients with suspected UTI by a private laboratory in Rio de Janeiro state in July 2019 and cultured onto Cystine Lactose Electrolyte Deficient agar (CLED) plates. Identification of non-duplicate isolates and antimicrobial susceptibility testing were performed by the VITEK®2 automated system (bioMérieux, Marcy l'Etoile, France). *E. coli* isolates with bacterial counts ≥10^5^ CFU/mL were sent to our laboratory with VITEK®2 reports and patient demographic data.

### Bacterial identification

We reassessed bacterial species by mass spectrometry with matrix-assisted laser desorption ionization-time of flight (MALDI-TOF, Bruker Biotyper 3.1, Bruker Daltonics, Billerica, MA). Protein mass spectrum was obtained with the flexControl software, v3.4 (Bruker Daltonics, EUA), and compared to MALDI Biotyper v3.1 (Bruker Daltonics) database. Score values of 2,000 or higher were considered as species confirmation and *E. coli* isolates were stored in skim milk with 10 % glycerol at -20 °C.

### Antimicrobial susceptibility

Data obtained by VITEK®2 automated system included susceptibility to amoxicillin-clavulanic acid, ampicillin, amikacin, ciprofloxacin, cefuroxime, gentamicin, meropenem, nitrofurantoin, norfloxacin and trimethoprim-sulfamethoxazole. We detected ESBL production by a double-disk synergy test with aztreonam, cefepime, cefotaxime and ceftazidime discs at a distance up to 2.5 cm from center to center to an amoxicillin-clavulanic acid disc.^24^ We defined as MDR all isolates non-susceptible to one or more agents in three or more antimicrobial classes.^25^

### Strain typing

ESBL-producing isolates were typed by MLST with the Achtman scheme.^26^ DNA sequences were determined and compared with references (https://pubmlst.org/). We identified ESBL-encoding genes by PCR^27^ and determined *bla*_CTX-M_ groups 1, 2, 8 and 9 sequences for analyses with BioEdit v7.2.5 and comparisons by BLAST with data available at the NCBI database. The whole collection was analyzed by PCR screening for the *E. coli* pandemic lineages ST131, ST69, ST73, and S95.^28^ The ST131 set was typed for clades and subclades by PCR,^29^ and *fimH* alleles by PCR screening,^30^ sequencing to assess clade-specific single nucleotide polymorphisms at the FimTyper 1.0 platform (https://cge.cbs.dtu.dk/services/FimTyper/).

### Statistical analysis

Antimicrobial resistance frequencies are described as counts and percentages and compared with data from a previous study with isolates obtained from outpatients by the same laboratory and geographic region in 2015.^11^ Univariate analyses of the distribution of resistance to each agent, MDR and ESBL production by gender and age were performed with the Chi-Square or Fisher's exact tests. Bonferroni post-hoc test was used to compare the results between age strata. We used SPSS® Statistics V.23 for statistical analysis and defined significance for two-tailed p-value < 0.05.

### Ethics

The study was approved by the Human Research Ethics Committee of Universidade Federal do Rio de Janeiro (#CAEE 59953322.9.0000.5257).

## Results

**Fig. 1:**
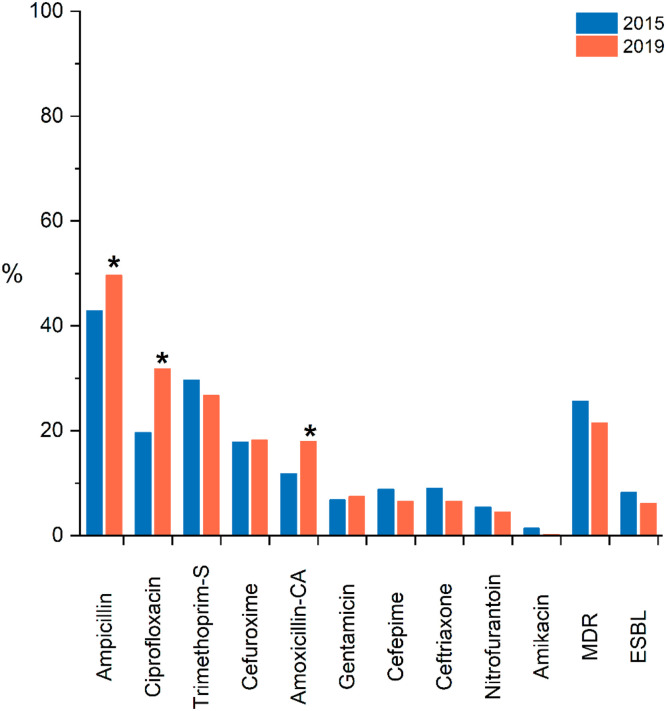
Frequencies of antimicrobial non-susceptible *E. coli* isolates among isolates obtained in 2015 and 2019. * Indicates a significant difference (p < 0.05); S, Sulfamethoxazole; CA, Clavulanic Acid.

We included 992 confirmed *E. coli* isolates in the study, each from a different subject. Antimicrobial susceptibility results obtained by the VITEK system were available for 982 (99 %) isolates, described in Fig. 1, together with data from 499 *E. coli* isolates from the same study population obtained in 2015.^11^ Resistance was higher than 30 % for ampicillin and ciprofloxacin, with a significant increase for these two drugs compared with 2015.^11^ Resistance to gentamicin, nitrofurantoin, and amikacin remained less than 8 % and was not detected for meropenem. A total of 62 (6 %) isolates produced ESBL and 210 (21 %) were MDR, without statistically significant differences over time.

About 95 % of subjects were female. Resistance rates were usually higher for isolates from men, with a statistically significant difference for ESBL production (14 % vs. 6 %, p = 0.04), as shown in Table 1. Subjects older than 59-years were the predominant age group (50 %) and had significantly higher resistance rates (with few exceptions for antimicrobial agents where resistance was rare and for sulfamethoxazole-trimethoprim). The proportion of isolates that were MDR or ESBL producers was also significantly higher in this age group, as shown in Table 2. ESBL-producing isolates carried a *bla*_CTX-M_ type gene, mainly *bla*_CTX-M15_ (n = 30, 48 %) (Table 3), and were mostly ST131 clone (n = 32, 52 %).

**Table 1:** Distribution of antimicrobial non-susceptible *E. coli* isolates by gender.

**Antimicrobial agent**	**Number and (%)**	
	**Female** **(n** = **931)**	**Male** **(n** = **51)**
Amikacin	3 (0.3)	0
Amoxicillin-clavulanic acid	165 (18)	13 (26)
Ampicillin	463 (50)	26 (51)
Cefepime	59 (6)	7 (14)
Ceftriaxone	59 (6)	7 (14)
Cefuroxime	166 (18)	15 (29)
Ciprofloxacin	292 (31)	22 (43)
Gentamicin	70 (8)	4 (8)
Nalidixic acid	368 (40)	29 (57)^a^
Nitrofurantoin	41 (4)	3 (6)
Piperacillin-tazobactam (n = 965)	7 (1)	0
Trimethoprim-sulfamethoxazole	250 (27)	13 (26)
ESBL	55 (6)	7 (14)^b^
MDR	197 (21)	13 (26)

a p = 0.02.

b p = 0.04. Other values are p > 0.05.

**Table 2:** Distribution of antimicrobial non-susceptible *E. coli* isolates by age range.

**Antimicrobial agent**	**Age range, n and (%)**				**p**
	**< 18** **(n** = **30)**	**18‒39** **(n** = **190)**	**40‒59** **(n** = **267)**	**> 59** **(n** = **495)**	
Amikacin	1 (3)	0 (0)	1 (0.4)	1 (0.2)	>0.05
Amoxicillin-clavulanic acid	6 (20)	25 (13)	37 (14)	110 (22)^a^	**<0.01**
Ampicillin	18 (60)	92 (48)	115 (43)	264 (53)^b^	**0.03**
Cefepime	3 (10)	8 (4)	7 (3)	48 (10)^b^	**<0.01**
Ceftriaxone	3 (10)	8 (4)	7 (3)	48 (10)^b^	**<0.01**
Cefuroxime	5 (17)	29 (15)	43 (16)	104 (21)	>0.05
Ciprofloxacin	6 (20)	39 (21)	80 (30)	189 (38)^c^	**<0.01**
Gentamicin	2 (7)	12 (6)	19 (7)	41 (8)	>0.05
Nitrofurantoin	1 (3)	5 (3)	12 (5)	26 (5)	>0.05
Piperacillin-tazobactam (n = 965)	0 (0)	1 (0.5)	2 (0.8)	4 (0.8)	>0.05
Trimethoprim-sulfamethoxazole	8 (27)	50 (26)	64 (24)	141 (29)	>0.05
ESBL	3 (10)	7 (4)	7 (3)	45 (9)^b^	**<0.01**
MDR	6 (20)	29 (15)	44 (17)	131 (27)^a^	**<0.01**

p-values in bold indicate a significant difference (p < 0.05), in post-hoc analysis, compared with ^a^ Subgroups 18‒39y and 40‒59y

b Subgroup 40‒59y, and ^c^ Subgroup 18‒39y.

**Table 3:** Clonal distribution and *bla*_CTX-M_ types of 62 ESBL -producing *E. coli* isolates from 2019.

**ST, n (%)**	**ESBL, n (%)**
ST131 (32, 52)	CTX-M-15, 19 (31); CTX-M-27, 7 (11); CTX-M-8, 2 (3); CTX-M-2, 1 (2); CTX-M-14, 1 (2); CTX-M-55, 1 (2); CTX-M-238, 1 (2)
ST1543 (4, 6)	CTX-M-14, 2 (3); CTX-M-8, 2 (3)
ST38 (2, 3)	CTX-M-15, 1 (2); CTX-M-27, 1 (2)
ST162 (2, 3)	CTX-M-15, 2 (3)
ST410 (2, 3)	CTX-M-15, 1 (2); CTX-M-8, 1 (2)
ST648 (2, 3)	CTX-M-15, 2 (3)
ST1163 (2, 3)	CTX-M-2, 2 (3)
ST1193 (2, 3)	CTX-M-27, 2 (3)
Other ST with one isolate each (14, 23)^a^	CTX-M-15, 5 (8); CTX-M-27, 2 (4); CTX-M-8, 7 (11)

a ST10, ST58, ST68, ST69, ST90, ST167, ST224, ST297, ST559, ST945, ST1308, ST1845, ST2873, ST10990.

**Fig. 2:**
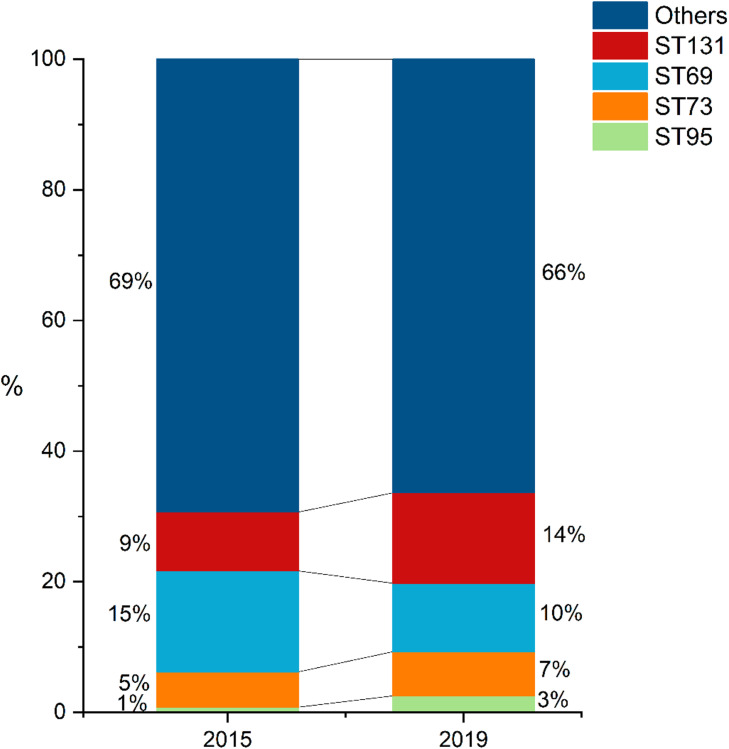
Frequencies of *E. coli* clones among isolates obtained in 2015 and 2019.

A total of 333 isolates were identified as one of the four pandemic lineages screened (Table 4). The combined participation of these clones increased in non-significant differences from 34 % in 2015 to 34 % in 2019 (Fig. 2). This trend was driven by the steadily increased frequency of ST131, from 9 % in 2015 to 14 % in 2019, with a statistically significant difference (p = 0.009) (Table 4). Noteworthy, isolates came from widely distributed collecting stations in the metropolitan area encompassing Rio de Janeiro and several other neighboring cities.

**Table 4:** Frequencies of *E. coli* clones in 2005, 2015 and 2019.

**ST**	**Number and (%) of isolates**	
	**2015 (n** = **499)**	**2019 (n** = **992)**
ST131	45 (9)^a^	137 (14)^a^
ST69	77 (15)	104 (11)^a^
ST73	27 (5)^a^	67 (7)
ST95	4 (1)	25 (3)^a^
Others	346 (69)	659 (66)

a Indicates a significant difference compared to previous period; S, Sulphamethoxazole; CA, Clavulanic Acid.

**Fig. 3:**
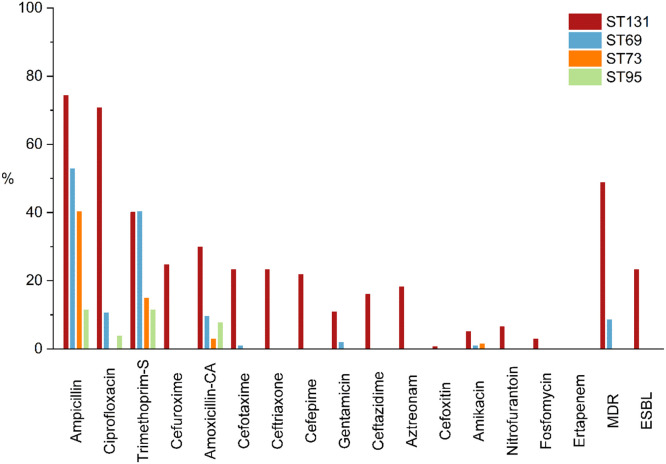
Frequencies of non-susceptible *E. coli* clones in 2019.

In 2019, resistance to antimicrobial agents was usually higher among ST131, reaching over 70 % for ampicillin and ciprofloxacin, and 49 % were MDR (Fig. 3). Most ST131 isolates were clade C (56 %, n = 76) (Table 5), a lineage with a significantly higher frequency of ESBL-producing isolates (33 %, n = 25/76) than among ST131-non-C isolates (12 %, n = 7/60, p = 0.007). However, within clade C, ESBL production was significantly higher in C2 (56 %, n = 18/33) and C1‒27 (78 %, n = 7/9) compared to C1 (0/34, p < 0.001). *fimH* alleles in clade C were mostly type 30 (89 %) and included two new types (H1590 and H3363), each with one different non-synonymous mutation compared to *fimH*30 (A115V and T40R, respectively). Alleles were all type 41 among ST131-A and were highly variable among ST131-B.

**Table 5:** Distribution clades and CTX-M types of 136 ST131 isolates.

**Clade (n)**	**Subclade (n)**	***fimH* type (n)**	**ESBL (n)**
A (21)	Not applicable	41 (21)	None (21)
B (39)	Not applicable	22 (15)	CTX-M-2 (1), CTX-M-55 (1), CTX-M-8 (1), none (12)
		64 (4)	None (4)
		27 (3)	CTX-M-15 (2), none (1)
		377 (3)	CTX-M-8 (1), none (2)
		5 (2)	CTX-M-14 (1), none (1)
		70 (2)	None (2)
		207 (2)	None (2)
		9, 20, 25, 30, 32, 47, 148, 298 (one each)	None (8)
C (76)	C2 (33)	30 (32)	CTX-M-15 (17), CTX-M-238 (1), none (14)
		None (1)	None (1)
	C1 (34)	30 (27)	None (27)
		27 (2)	None (2)
		32, 54, 137, 1590, 3363 (one each)	None (5)
	C1-M27 (9)	30 (9)	CTX-M-27 (7), none (2)
C0 (1)	Not applicable	30 (1)	None (1)

## Discussion

We report high antimicrobial resistance rates for commonly used drugs such as ampicillin and ciprofloxacin, among *E. coli* from ITU diagnosed in the community in Rio de Janeiro in 2019. These rates are even higher than observed in a similar study from 2015,^11^ posing an increasing challenge for the empiric treatment of UTI. Previous consensus guidelines had proposed resistance thresholds to help select appropriate drug prescriptions; however, an updated consensus suggests such limits are arbitrary, and a core international guideline should be developed with local adaptations based on regional resistance rates.^31^ In the present study resistance rates were higher in men and the elderly, calling for special attention for these groups.

Overall, ciprofloxacin resistance (32 %) showed a significant increase compared to 2015. Rates higher than 70 % were already reported in previous studies from Brazil, in the cities of Rio de Janeiro and Londrina with similar methodology.^11^^,^^32^ The increasing resistance to fluoroquinolones among human bacterial pathogens is a growing problem worldwide.^33^ Between 2016 and 2021, fluoroquinolone use decreased in both the community and hospital sectors in more than half of the European Union countries;^34^ unfortunately, in several European countries, the United States and Brazil, fluoroquinolones are still being prescribed for patients with uncomplicated UTI.^33^^,^^35^

In the present study, ESBL-producing isolates were 6 % of the whole collection, belonging to 22 ST. Among these, ST131 was by far the most frequent clone (52 %), with a significantly increased fraction compared to 2015 (21 %, p = 0.005).^11^ Well-described MDR clones (ST131, ST1193, ST410, and ST648) formed more than 60 % of ESBL isolates in 2019. ESBL-producing ST648 isolates have already been detected as a cause of fluoroquinolone-resistant community UTI^11^ and as a colonizer in wild birds,^36^ in Rio de Janeiro serving as a marker for environmental contamination of clinically relevant drug resistance genes in the city. ST1193 and ST410 were detected in a different study with human UTI,^23^ though isolates did not produce ESBL. The ST1193 clone has emerged as a cause of UTI and ICS associated with fluoroquinolone resistance worldwide over the past ten years^37^^,^^38^ and has a potential for rapid dissemination in Brazil incrementing the pool of MDR infections.

Here, all ESBL isolates carried a *bla*-_CTX-M_ type gene, as we observed in other community settings in Rio de Janeiro in UTI causing ESBL-*E. coli* from 2015,^11^ and in almost all (46 in 47) human gut colonizers detected in 2015‒2019.^39^ As expected, the *bla*-_CTX-M-15_ gene was the predominant subtype. These findings mirror the dissemination and predominance of CTX-M-type enzymes worldwide since the early 2000s.^40^

The four *E. coli* STs investigated in this study contained more than one-third of all isolates, showing the clonal nature of these infections, even in a community setting, as consistently described.^7^^,^^41^ The uneven distribution of resistance among these clones has been demonstrated in several surveillance studies.^9^, ^10^, ^11^^,^^13^^,^^42^^,^^43^ ST73 and ST95 are consistently highly susceptible, while ST69 and ST131 are major contributors to MDR infections. This same variability is seen within the ST131 clone, composed of sublineages A and B, highly susceptible, C1 with variable resistance profiles, and C2, predominantly ESBL positive and resistant to ciprofloxacin. Other studies conducted outside the USA and the European Union performed individual analyses of specific strains, particularly ST131. However, these reports did not aim to describe their overall frequency,^23^^,^^44^, ^45^, ^46^ preventing comparisons with data from the present study.

The composition of clones may change over time, with a potential impact on antimicrobial susceptibility rates. For example, in the present study, from 2015 and 2019, we detected a significant increase in the fraction of UTI caused by ST131 isolates in the community in Rio de Janeiro which encompassed 14 % of the isolates of the present collection. The high rate of resistance to ciprofloxacin (> 70 %), typical of this clone, had an important impact on the overall resistance to this drug, which increased from 20 %^11^ to 32 %. Moreover, over half of ESBL-producing isolates belonged to ST131. As expected, the relative frequencies of sublineages differed widely; most isolates were clade C (56 %) and H30 type (51 %). A single H30 isolate was clade B, while all others were C, indicating that the *fim*H-30 allele in ST131 is a marker of clade C. Expansion of ST131-H30 has been reported in previous studies and has been primarily justified by higher frequency of resistance and carriage of ESBL genes.^6^^,^^9^^,^^47^

The findings of the present study may have resulted from the selective pressure of antimicrobial agents, but research data has shown that additional features drive the establishment and expansion of these clones. Indeed, a large genomics-based molecular epidemiology study has shown that the benefits from positive selection by use were not uniform across different clones or classes of antibiotics.^9^ Unexpectedly, antibiotic use in higher amounts in different countries correlated with the expansion of MDR clones as well as non-MDR clones. Thus, the forces driving the emergency and evolution of a pandemic clone are still not well understood. One proposed mechanism of stability of a clone independent of resistance is multilocus Negative Frequency-Dependent Selection (NFDS),^48^ formed by rare phenotypes among a population, which could take advantage of characteristics such as a new antigen or resource-use strategy. These phenotypes would be less costly for the bacterial population, less recognized by the host, and could provide less competition for the same resources among isolates. Surveillance, preferably with high-resolution techniques and fitness studies, is a critical tool for understanding the dynamics of antimicrobial resistance, and *E. coli* is a convenient sentinel organism for this purpose.

One limitation of the present study is the lack of data about antimicrobial consumption and health status from all participants. Such information was unavailable for the project due to the absence of a central medical record database for outpatients in the city. However, we believe that results illustrate clearly the severe stage of antimicrobial resistance and the emergency of ST131 as a dominant cause of resistant UTI.

In conclusion, in the present study, from 2015 and 2019, we detected a significant increase in the fraction of UTI caused by ST131 isolates in the community in Rio de Janeiro with an overall rise in ciprofloxacin resistance. This clone, especially Clade C2, corresponded to more than half the ESBL producers. The impact of ST131 on antimicrobial resistance and ESBL production is remarkable, and the dissemination of this clone is a public health concern. In addition, early identification of high impact MDR clones may help prevent severe patient outcomes and reduce healthcare costs. The findings of this study may contribute to a global understanding of *E. coli* antimicrobial resistance evolution, especially in low- and middle-income countries, where data on the prevalence of these pandemic clones remains scarce. Elucidating antimicrobial resistance patterns of these highly disseminated clones may help to guide new protocols for initial treatment of UTI and other *E. coli* infections. As a sentinel organism, monitoring the dissemination of antimicrobial-resistant pandemic clones may offer insights into similar dynamics in other microorganisms, particularly *Enterobacteriaceae*. Further approaches may include other protocols to identify clones not screened in this study with an impact on overall resistance.

## Conflicts of interest

The authors declare no conflicts of interest.
